# Dietary Fiber Is Inversely Associated with Central Arterial Stiffness Progression, While Alcohol and Iron Intake Are Positively Associated with CAVI: A 5-Year Longitudinal Study

**DOI:** 10.3390/nu18091314

**Published:** 2026-04-22

**Authors:** Javier Alonso-Diaz, Marta Gómez-Sánchez, David Arjol, Susana Gonzalez-Sánchez, Emiliano Rodríguez-Sánchez, Luis García-Ortiz, Leticia Gómez-Sánchez, Manuel A. Gómez-Marcos, EVA Investigators Group

**Affiliations:** 1 Primary Care Research Unit of Salamanca (APISAL), Avd. Portugal, 37005 Salamanca, Spain; jalonsodiaz@usal.es (J.A.-D.); martagmzsnchz@gmail.com (M.G.-S.); darjol@ibsal.es (D.A.); gongar04@gmail.com (S.G.-S.); emiliano@usal.es (E.R.-S.); lgarciao@usal.es (L.G.-O.); leticiagmzsnchz@gmail.com (L.G.-S.); 2 Institute of Biomedical Research of Salamanca (IBSAL), Paseo de San Vicente, 37007 Salamanca, Spain; 3 Home Hospitalization Service, Marqués of Valdecilla University Hospital, 39008 Santander, Spain; 4 Research Network on Chronicity, Primary Care and Health Promotion (RICAPPS), 37005 Salamanca, Spain; 5 Faculty of Nursing and Physiotherapy, University of Salamanca, Avenida de los Donantes de Sangre, s/n, 37008 Salamanca, Spain; 6 Salamanca Primary Care Management, Castilla and León Health Service–SACYL, 37005 Salamanca, Spain; 7 Department of Medicine, University of Salamanca, Av. Campo Charro, 37007 Salamanca, Spain; 8 Department of Biomedical and Diagnostic Sciences, University of Salamanca, Av. Campo Charro, 37007 Salamanca, Spain; 9 Emergency Service, University Hospital of La Paz. P. of Castellana, 261, 28046 Madrid, Spain

**Keywords:** arterial stiffness, diet, macronutrients, minerals, carotid–femoral pulse wave velocity, cardio-ankle vascular index

## Abstract

**Background**: Arterial stiffness (AS) is a key marker of vascular aging and an independent predictor of cardiovascular risk. Although diet has been proposed as an important modifiable factor influencing vascular health, the independent associations between specific macronutrients and minerals and the progression of arterial stiffness remain insufficiently characterized. **Objective**: The aim of this longitudinal study was to evaluate the independent associations of baseline dietary macronutrient and mineral intake with the 5-year progression of arterial stiffness (assessed via carotid–femoral pulse wave velocity (cfPWV) and cardio-ankle vascular index (CAVI) in adults without prior cardiovascular disease. **Methods**: This longitudinal study included 466 participants from the EVA study who were evaluated at baseline and after a five-year follow-up (mean age 55.96 ± 14.15 years; 51.1% women). Arterial stiffness was assessed using cfPWV and CAVI. Dietary intake of macronutrients and minerals was estimated using the EVIDENT smartphone application. Multivariable linear regression models were used to examine the association between nutrient intake and arterial stiffness progression. Model 1 was adjusted for age and sex, and Model 2 was additionally adjusted for lifestyle variables and cardiovascular risk factors. Dietary intake was exclusively documented at baseline using a 3-day dietary record, while arterial stiffness parameters (cfPWV and CAVI) were assessed both at baseline and at the five-year follow-up. **Results**: Higher dietary fiber intake was independently associated with a lower increment in cfPWV after full adjustment (β = −0.025; 95% CI (confidence interval): −0.046 to −0.005). Alcohol intake showed a positive association with CAVI increment in the fully adjusted model (β = 0.020; 95% CI: 0.006 to 0.034). Iron intake was also independently associated with increased CAVI (β = 0.022; 95% CI: 0.004 to 0.041). Carbohydrate intake showed a small positive association with CAVI, whereas no consistent independent associations were observed for other macronutrients or minerals. **Conclusions**: In this adult population without previous cardiovascular disease, higher dietary fiber intake was associated with lower progression of central arterial stiffness, whereas alcohol and iron intake showed positive associations with peripheral arterial stiffness. Overall, most nutrients were not independently related to arterial stiffness after comprehensive adjustment. These findings suggest that selected dietary components may contribute modestly to vascular aging.

## 1. Introduction

Cardiovascular diseases remain the leading cause of mortality worldwide, accounting for nearly a third of all global deaths [1]. In this context, premature vascular aging is emerging as a major public health challenge, affecting a substantial proportion of the adult population even prior to the onset of clinical symptoms [2]. Arterial stiffness is a central manifestation of vascular aging and an independent predictor of cardiovascular events [3,4]. To achieve a comprehensive assessment of the arterial tree, it is necessary to combine parameters that evaluate central arterial stiffness, such as carotid–femoral pulse wave velocity (cfPWV), the gold standard for aortic stiffness, with parameters that also assess peripheral arterial stiffness, such as the cardio-ankle vascular index (CAVI), which provides a complementary measure of the arterial tree with less dependence on blood pressure (BP) [5,6,7,8,9,10]. Together, these allow early detection of subclinical vascular damage.

Diet is one of the main modifiable determinants of vascular health; however, its specific role in the progression of arterial stiffness remains incompletely understood [11,12,13]. While healthy dietary patterns have been associated with more favorable vascular profiles [14,15,16,17], the independent longitudinal impact of specific macronutrient and mineral intake requires further clarification. Observational evidence suggests that dietary composition directly influences vascular function; for example, refined carbohydrates, simple sugars, and saturated fats may accelerate vascular aging through increased systemic inflammation and endothelial dysfunction [18,19,20]. In contrast, dietary fiber exerts protective vascular effects [21,22,23,24,25]. In addition, specific minerals play a relevant role in vascular oxidative stress: alterations in iron metabolism may promote endothelial damage [26,27,28,29,30]; magnesium acts as a key cofactor in nitric oxide production and as a natural calcium antagonist, preventing medial calcification and promoting arterial distensibility [31,32]; and inadequate intake of zinc and selenium increases oxidative stress and low-grade inflammation, promoting the transition from an elastic to a stiff vascular phenotype [28]. Meanwhile, excessive alcohol consumption contributes to increased blood pressure and arterial stiffness [33,34,35,36,37].

Despite this biological plausibility, few population-based studies have simultaneously evaluated the longitudinal impact of macronutrient and mineral intake using complementary measures of arterial stiffness (cfPWV and CAVI). Moreover, although we have recently documented longitudinal associations between the intake of specific vitamins and arterial stiffness in this cohort [38], the independent predictive value of macronutrients and essential minerals over a 5-year follow-up remains to be fully elucidated. In this context, jointly evaluating macronutrient and mineral intake using both cfPWV and CAVI in a longitudinal design may provide a more comprehensive understanding of the role of diet in vascular aging. Therefore, the primary objective of this longitudinal study was to evaluate the association between baseline dietary macronutrient and mineral intake and the 5-year progression of arterial stiffness, measured using cfPWV and CAVI, in a cohort of adults without previous cardiovascular disease. Understanding the specific role of these dietary components in the trajectory of vascular aging over time may contribute to identifying modifiable lifestyle factors crucial for early cardiovascular prevention.

## 2. Materials and Methods

### 2.1. Experimental Approach and Ethical Considerations

This was a population-based study with both cross-sectional and longitudinal analyses conducted within the framework of the Association between different risk factors and vascular accelerated aging (EVA study; NCT02623894) [39]. The study population consisted of individuals receiving care in five urban primary care centers. Participants were selected through stratified random sampling with replacement according to age groups (35, 45, 55, 65, and 75 years) and sex. From a reference population of 43,946 individuals, a total of 501 participants were included, approximately 100 per age group, with a balanced distribution of men and women. Participants aged 35 and older were originally selected for the EVA study [39], as clinically detectable and progressive changes in arterial stiffness typically begin to manifest in the third or fourth decade of life, ensuring measurable progression over a 5-year follow-up period. Baseline assessment and participant recruitment were carried out between June 2016 and November 2017. The follow-up visit was conducted approximately five years later, between May 2021 and October 2022. Inclusion criteria were age between 35 and 75 years and provision of written informed consent. Exclusion criteria included terminal illness, inability to attend the health center, previous cardiovascular disease, estimated glomerular filtration rate below 30 mL/min/1.73 m^2^, chronic inflammatory diseases or acute inflammatory processes within the previous three months, and treatment with estrogens, testosterone, or growth hormone. At the five-year follow-up evaluation, 480 participants were reassessed. The final analytical sample consisted of 466 individuals who completed both evaluations and had complete three-day dietary records. Detailed inclusion and exclusion criteria, as well as flow diagrams illustrating the initial recruitment algorithm and the 5-year follow-up process (including losses), are provided in Figures S1 and S2 (Supplementary Material). The study was conducted and reported in accordance with the Strengthening the Reporting of Observational Studies in Epidemiology (STROBE) guidelines [40] (see the complete checklist in Supplementary Material Table S1).

The study was approved by the Drug Research Ethics Committee of the Salamanca Health Area on 4 May 2015 for the baseline evaluation and on 13 November 2020 for the follow-up assessment (reference code PI 2020 10 569). All participants provided written informed consent prior to their inclusion in the study. The research was conducted in accordance with the ethical principles established in the Declaration of Helsinki [41]. Data were anonymized before analysis through the assignment of unique alphanumeric identifiers. Access to personal information was restricted to authorized members of the research team.

### 2.2. Arterial Stiffness Measurements

Central arterial stiffness was assessed using carotid–femoral pulse wave velocity (cfPWV) measured with the SphygmoCor system (AtCor Medical Pty Ltd., West Ryde, Australia). Pulse waves were recorded at the carotid and femoral arteries with the participant in the supine position. Transit time was estimated relative to the R wave of the electrocardiogram. Distances were measured with a tape measure from the suprasternal notch to the carotid and femoral recording sites. cfPWV was calculated according to established measurement recommendations [42].

Peripheral arterial stiffness was assessed using the cardio-ankle vascular index (CAVI) measured with the VaSera VS-1500 device (Fukuda Denshi Co., Ltd., Tokyo, Japan), following the manufacturer’s instructions. For the measurement, cuffs were placed on both arms and ankles [43].

To minimize the influence of acute factors on vascular measurements, participants were instructed to avoid caffeine consumption, smoking, and vigorous physical activity for at least three hours prior to the assessment. Additionally, they remained at rest for a minimum of five minutes before measurements were performed.

### 2.3. Assessment of Macronutrient and Mineral Intake

Dietary intake was assessed using a three-day food record collected through the EVIDENT mobile application. This application was developed and validated by the Primary Care Research Group of Castilla y León (REDIAPP) and is registered under intellectual property number 00/2014/2207 [44]. The dietary assessment tool was designed to record foods and beverages, but not to systematically capture the use of vitamin or mineral supplements. To accurately capture the natural variability in eating habits throughout the week, participants were instructed that the 3-day dietary record must systematically include two weekdays and one weekend day. Participants recorded all foods and beverages consumed over three consecutive days, including portion size and preparation method. Foods were classified into predefined categories within the application.

Daily intake of macronutrients (energy, protein, carbohydrates, total fat, saturated fatty acids, monounsaturated fatty acids (MUFAs), polyunsaturated fatty acids, dietary fiber, cholesterol, alcohol, and water) and minerals (iron, iodine, magnesium, zinc, selenium, sodium, and potassium) was estimated using Spanish food composition tables. Alcohol intake was estimated exclusively from beverages reported in the 3-day dietary record, calculating the average daily intake in grams. No specific validated questionnaires for alcohol consumption were administered.

### 2.4. Sociodemographic Variables, Lifestyle Factors, and Laboratory Analyses

Sociodemographic and lifestyle variables were collected using standardized questionnaires. Age and sex were recorded for all participants. Lifestyle-related factors included smoking status and alcohol consumption. Adherence to the Mediterranean dietary pattern was assessed using the 14-item Mediterranean Diet Adherence Screener (MEDAS) [45]. Sedentary behavior was assessed using the Marshall Sitting Questionnaire [46,47], and physical activity was measured using the short version of the International Physical Activity Questionnaire (IPAQ-SF) [48,49], with results expressed in metabolic equivalent task minutes per week (MET-min/week). Fasting blood samples were obtained to determine lipid profile and plasma glucose levels. Blood pressure and heart rate were measured using a validated automated sphygmomanometer (OMRON M10-IT (OMRON Healthcare, Kyoto, Japan)) following the recommendations of the European Society of Hypertension [50].

### 2.5. Statistical Analysis

Statistical analyses were performed using IBM SPSS Statistics version 28.0 (IBM Corp., Armonk, NY, USA). All tests were two-tailed, and statistical significance was set at *p* < 0.05. Descriptive Analysis: The normality of continuous variables was assessed using the Shapiro–Wilk test. Skewness and kurtosis values were also examined to characterize data distribution. Variables with approximately normal distribution were expressed as mean ± standard deviation (SD), whereas variables with non-normal distribution were described as median and interquartile range (IQR). Comparisons between men and women were performed using Student’s *t*-test for independent samples when normality assumptions were met. Otherwise, the Mann–Whitney U test was applied. Given the sample size (*n* = 466), parametric tests were considered robust to moderate deviations from normality. To control for multiple comparisons in nutritional analyses, *p*-values were additionally adjusted using the Benjamini–Hochberg false discovery rate (FDR) procedure. Effect sizes were calculated for all between-group comparisons. Cohen’s d was used for parametric tests and the r statistic for non-parametric tests. Ninety-five percent confidence intervals (CIs) were estimated for effect size measures.

Multivariable Analysis: Multivariable linear regression models were used to examine the association between dietary intake (macronutrients and minerals) and arterial stiffness progression. In these models, arterial stiffness progression (defined as the 5-year changes in cfPWV and CAVI) was considered the dependent variable, while dietary intake of macronutrients and minerals was included as the main independent variable. Two hierarchical models were fitted for each dependent variable: Model 1 was adjusted for age and sex. Model 2 was additionally adjusted for potential confounding factors, including total energy intake, Mediterranean diet adherence score, physical activity, smoking status, alcohol consumption, body mass index (BMI), pulse pressure, heart rate, LDL (low-density lipoprotein) cholesterol, fasting glucose, and baseline arterial stiffness. Covariates were selected a priori based on their clinical relevance and potential role as confounders in the relationship between diet and arterial stiffness. Dietary variables were introduced as continuous predictors and entered separately into independent models to prevent collinearity. Model assumptions, including normality of residuals and homoscedasticity, were verified. Multicollinearity among covariates was ruled out using the Variance Inflation Factor (VIF), with all VIF values being <2.0. Regression coefficients (β) and their corresponding 95% confidence intervals were reported. The assumptions of linear regression, including linearity and homoscedasticity of residuals, were evaluated through graphical inspection. Given the observational and exploratory nature of the nutrient-specific analyses, no formal correction for multiple comparisons was applied in the multivariable models, aiming to minimize Type II errors in identifying potentially relevant clinical signals.

## 3. Results

### 3.1. Clinical and Vascular Characteristics

The baseline clinical, lifestyle, and vascular characteristics of the study population are summarized in Table 1. Overall, men and women showed differences in several behavioral and cardiometabolic variables, including alcohol consumption, physical activity, sedentary time, and blood pressure. Arterial stiffness parameters were slightly higher in men, although these differences were modest.

Detailed information on variable distributions and effect sizes is provided in the Supplementary Material (Tables S2 and S3).

### 3.2. Macronutrient Intake

Macronutrient intake according to sex is presented in Table 2. Significant baseline sex differences were observed in the absolute intake (grams/day) of several macronutrients, including total proteins, total fats, and specific fatty acids (SFA, MUFA, PUFA), with men showing consistently higher absolute intakes than women. However, this was largely driven by their higher total energy intake. When evaluating the macronutrient distribution as a percentage of total energy intake, these differences disappeared. The relative contribution of proteins, carbohydrates, and total fats to the overall energy intake was highly comparable between men and women, reflecting a similar underlying dietary pattern despite differences in absolute volume. Men showed higher overall energy intake and greater consumption of several macronutrients, particularly fat-related components and alcohol, whereas dietary fiber intake was similar between sexes.

Further details regarding distributional properties and effect sizes are provided in the Supplementary Material (Tables S4 and S5).

### 3.3. Mineral Intake

Significant baseline sex differences were observed in the absolute intake of several minerals; specifically, men exhibited higher intakes of Iodine, Zinc, Selenium and Nadium compared to women (Table 3). Additional information is available in the Supplementary Material (Tables S6 and S7).

### 3.4. Association Between Macronutrient Intake and Arterial Stiffness

**Figure 1:**
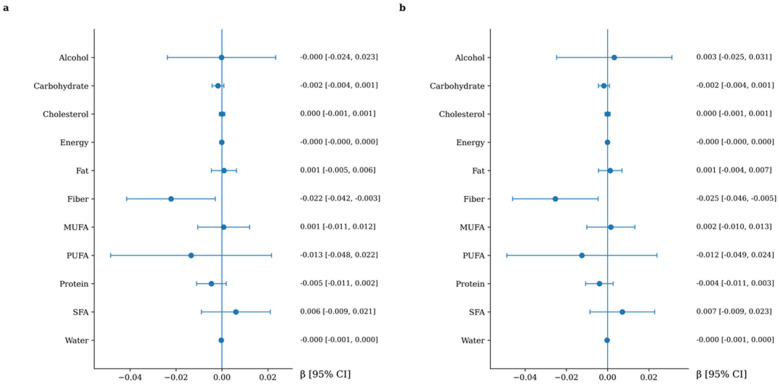
Association between macronutrient intake and cfPWV increment. Panel (**a**): adjusted for age and sex. Panel (**b**): additionally, adjusted for lifestyle variables and cardiovascular risk factors, including Mediterranean diet adherence score, physical activity, alcohol consumption, smoking status, body mass index, pulse pressure, heart rate, LDL cholesterol, and fasting glucose. Variables are listed in alphabetical order to facilitate quick reference.

**Figure 2:**
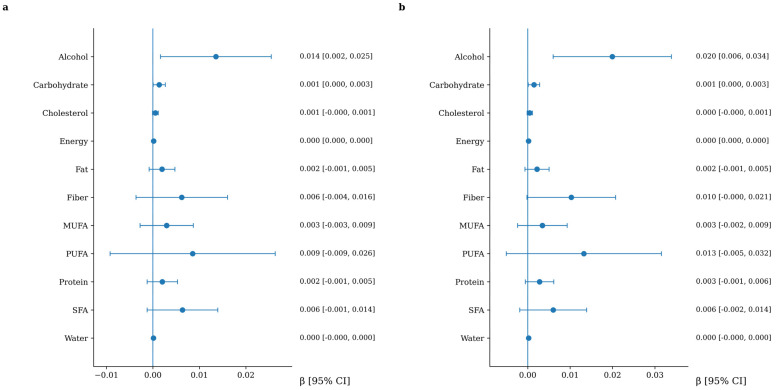
Association between macronutrient intake and CAVI increment. Panel (**a**): adjusted for age and sex. Panel (**b**): additionally, adjusted for lifestyle variables and cardiovascular risk factors, including Mediterranean diet adherence score, physical activity, alcohol consumption, smoking status, body mass index, pulse pressure, heart rate, LDL cholesterol, and fasting glucose. Variables are listed in alphabetical order to facilitate quick reference.

The longitudinal associations between macronutrient intake and changes in arterial stiffness are presented in Figure 1 and Figure 2. Higher dietary fiber intake was consistently associated with a lower progression of arterial stiffness, as assessed by cfPWV, and this association remained after multivariable adjustment. When expressed in clinically meaningful terms, each 10 g/day increase in dietary fiber intake was associated with a 0.25 m/s lower increase in cfPWV over follow-up (95% CI: −0.46 to −0.05). Regarding CAVI, alcohol intake showed a positive and independent association with arterial stiffness progression in fully adjusted models. When expressed in clinically meaningful terms, each 10 g/day increase in alcohol intake (approximately equivalent to one standard drink) was associated with an increase of 0.20 in CAVI over follow-up (95% CI: 0.06 to 0.34).

### 3.5. Association Between Mineral Intake and Arterial Stiffness

**Figure 3:**
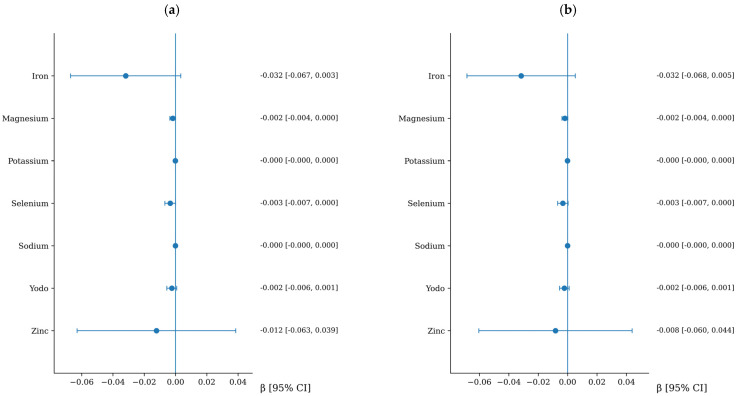
Association between mineral intake and cfPWV increment. Panel (**a**): adjusted for age and sex. Panel (**b**): additionally adjusted for lifestyle variables and cardiovascular risk factors, including Mediterranean diet adherence score, physical activity, alcohol consumption, smoking status, body mass index, pulse pressure, heart rate, LDL cholesterol, and fasting glucose. Variables are listed in alphabetical order to facilitate quick reference.

**Figure 4:**
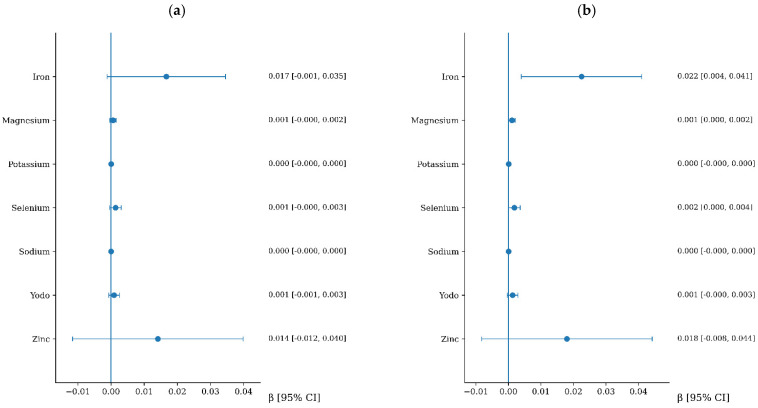
Association between mineral intake and CAVI increment. Panel (**a**): adjusted for age and sex. Panel (**b**): additionally, adjusted for lifestyle variables and cardiovascular risk factors, including Mediterranean diet adherence score, physical activity, alcohol consumption, smoking status, body mass index, pulse pressure, heart rate, LDL cholesterol, and fasting glucose. Variables are listed in alphabetical order to facilitate quick reference.

**Figure 5:**
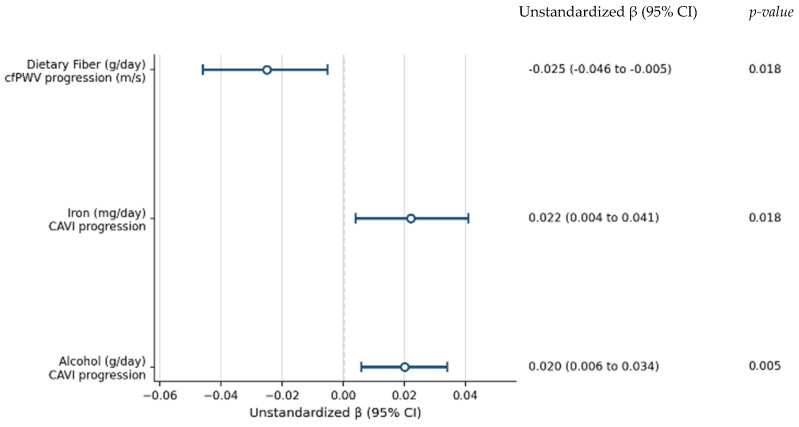
Independent associations between dietary fiber, alcohol, and iron intake and 5-year arterial stiffness progression. Forest plot showing unstandardized β coefficients and 95% confidence intervals from fully adjusted models.

The associations between mineral intake and arterial stiffness are presented in Figure 3 and Figure 4. No significant associations were observed between mineral intake and cfPWV progression. In contrast, iron intake was positively associated with CAVI progression after multivariable adjustment. When expressed in clinically meaningful terms, each 5 mg/day increase in iron intake was associated with an increase of 0.11 in CAVI over follow-up (95% CI: 0.02 to 0.21). For clarity, a summary of the associations that remained statistically significant in the fully adjusted models is presented in Table 4 and Figure 5. Furthermore, a sensitivity analysis was performed excluding extreme outliers (>3 standard deviations from the mean for nutrient intake and arterial stiffness progression). The exclusion of these outliers did not substantially alter the results, and the independent associations for dietary fiber, iron, and alcohol remained statistically significant, supporting the robustness of our models.

## 4. Discussion

### 4.1. Main Findings

In this five-year longitudinal study of adults without previous cardiovascular disease, we examined whether habitual intake of macronutrients and minerals was associated with the progression of arterial stiffness assessed with two complementary vascular markers, cfPWV and CAVI. The main findings were that higher dietary fiber intake was independently associated with a lower increase in cfPWV, whereas alcohol intake and iron intake were positively associated with CAVI progression. By contrast, most of the remaining nutrients were not independently associated with arterial stiffness after adjustment for lifestyle variables and cardiovascular risk factors. Overall, these results suggest that the relationship between diet and vascular aging is selective rather than generalized, and that it may differ according to the arterial stiffness index considered.

Interestingly, our baseline descriptive analysis revealed that while men consumed higher absolute amounts of macronutrients and overall energy, the relative macronutrient distribution (% of total energy) was comparable between sexes, suggesting a similar baseline dietary pattern in this cohort. An additional aspect that deserves consideration the role of chronological age in the interpretation of our findings. As expected, age is the main physiological driver of arterial stiffness [7]. However, the concept of early vascular aging (EVA) postulates that modifiable environmental factors may alter this natural trajectory. In our longitudinal analysis, the associations between specific dietary components—such as fiber, alcohol, and iron—and the progression of arterial stiffness remained significant after strict adjustment for chronological age. This suggests that dietary exposures may contribute to modulating vascular aging beyond the effect of time alone, either accelerating or attenuating arterial stiffening, and thus may play a role in defining the EVA phenotype.

A relevant aspect of the present findings is that the observed associations were not identical for cfPWV and CAVI. This distinction is important because cfPWV mainly reflects stiffness of the central elastic arteries, especially the aorta, whereas CAVI captures stiffness across a broader arterial segment extending from the aorta to peripheral muscular arteries and is less dependent on blood pressure at the time of measurement [5,8,9,10]. Thus, the differential associations observed in our study may indicate that specific dietary components influence distinct domains of the vascular aging process.

### 4.2. Macronutrients and Arterial Stiffness Progression

The most consistent macronutrient-related finding was the inverse association between dietary fiber intake and cfPWV progression. This result is coherent with previous longitudinal and observational evidence suggesting that higher fiber intake is associated with a more favorable vascular profile and lower arterial stiffness [21,22,25,51,52,53]. In particular, lower cumulative fiber intake across the life course has been linked to greater arterial stiffness in adulthood [21]. Several mechanisms could explain this association. Dietary fiber may improve lipid metabolism, attenuate postprandial glycemic responses, reduce systemic inflammation, and enhance the production of short-chain fatty acids through colonic fermentation, thereby contributing to better endothelial function and reduced arterial remodeling [23,24,25]. Within this framework, our findings support the possibility that fiber intake may exert a modest protective role against progression of central arterial stiffness even in adults without established cardiovascular disease.

A small positive association between carbohydrate intake and CAVI progression was observed after full adjustment. Although the magnitude of this relationship was limited, the finding remains of interest because it suggests that certain aspects of carbohydrate intake may contribute to peripheral or systemic arterial stiffening. Previous evidence is mixed. Some studies in populations at elevated cardiovascular risk have reported that higher carbohydrate intake is associated with poorer vascular function or increased arterial stiffness [19], but these associations are often difficult to interpret because the vascular effects of carbohydrates depend strongly on nutritional quality. Total carbohydrate intake does not distinguish between refined carbohydrates and high-quality carbohydrate sources rich in fiber and low glycemic load [54,55,56]. Therefore, the positive association observed here may reflect differences in carbohydrate quality rather than carbohydrate quantity alone. This interpretation is also consistent with the opposite direction observed for dietary fiber, suggesting that the source and composition of carbohydrate-containing foods may be more relevant than total intake itself.

Alcohol intake was positively associated with CAVI progression and remained significant after multivariable adjustment. This result is biologically plausible and broadly aligns with evidence indicating that alcohol consumption, especially at higher levels, is related to elevated blood pressure, oxidative stress, autonomic dysregulation, and endothelial dysfunction [33,34,35,57]. A recent systematic review concluded that the association between alcohol intake and arterial stiffness in healthy adults is heterogeneous, although higher consumption generally tends to be associated with a less favorable vascular profile [36]. Prospective evidence has also linked long-term alcohol consumption trajectories with arterial aging [58]. In our study, the association emerged for CAVI rather than cfPWV, which may indicate that alcohol-related vascular effects are more readily detected in arterial territories extending beyond the central aorta. However, the absence of detailed information on drinking patterns, including frequency and binge drinking, limits mechanistic interpretation.

The remaining macronutrients, including total fat, saturated fatty acids, polyunsaturated fatty acids, protein, cholesterol, and water intake, were not independently associated with arterial stiffness progression after full adjustment. This lack of association should be interpreted cautiously. It may reflect genuinely small independent effects, but it may also indicate that the vascular impact of these nutrients is mediated by broader metabolic pathways or is more appropriately captured at the level of dietary patterns rather than isolated nutrient totals. In this regard, monounsaturated fatty acids deserve particular mention. In Mediterranean populations, MUFA intake is often considered a marker of olive oil consumption and adherence to healthier dietary habits. Although MUFA intake was not independently associated with arterial stiffness progression in our models, this null finding does not necessarily contradict the broader evidence supporting favorable vascular effects of Mediterranean dietary patterns [12,13,14,17]. Rather, it suggests that the benefits of olive oil or MUFA-rich foods may be difficult to isolate when nutrients are analyzed individually and may depend on the broader dietary matrix in which they are consumed.

In addition, it should be noted that in our Mediterranean cohort, the main dietary source of monounsaturated fatty acids is olive oil, particularly extra virgin olive oil [16]. Therefore, the associations—or lack thereof—observed between total MUFA intake and arterial stiffness progression in our analysis inherently reflect the consumption of this key cardioprotective food, despite the fact that specific food groups were not isolated in the present nutrient-based models

### 4.3. Minerals and Arterial Stiffness Progression

Among minerals, the most notable finding was the positive association between iron intake and CAVI progression after full adjustment. This result is of particular interest because it was the only clear mineral signal that remained statistically significant in the fully adjusted models. The biological plausibility of this association is supported by evidence linking altered iron metabolism with oxidative stress, mitochondrial dysfunction, lipid peroxidation, endothelial damage, and arterial remodeling [28,29,30,59]. Observational studies have also reported associations between higher serum ferritin levels and increased arterial stiffness [26,27]. Nevertheless, this finding should be interpreted with caution. Dietary iron intake is not equivalent to systemic iron status, and our study did not distinguish between heme and non-heme iron or include biomarkers such as ferritin or transferrin saturation. Consequently, the observed association should be regarded as exploratory and hypothesis-generating.

In addition, it is important to recognize that the pro-oxidant vascular effects of dietary iron are likely to interact with the overall antioxidant status of the individual. While the potential protective role of antioxidant-rich nutrients has been addressed in previous analyses of this cohort [38], the balance between pro-oxidant minerals such as iron and dietary antioxidants may represent a key modulator of the vascular aging process.

The other minerals evaluated, including sodium, potassium, magnesium, iodine, zinc, and selenium, were not independently associated with progression of cfPWV or CAVI. This may appear unexpected, especially in the case of sodium and potassium, given previous reports linking these minerals to vascular structure and function [15,60,61]. However, several explanations are possible. First, the vascular effects of these minerals may depend more on long-term balance, urinary excretion, or dietary patterns than on short-term self-reported intake. Second, their effects may be partially mediated by blood pressure, which was included in the adjusted models and may therefore have attenuated direct associations. Third, in a relatively healthy adult population without previous cardiovascular disease, the independent contribution of individual minerals may be modest and difficult to detect after accounting for broader cardiovascular risk factors. In this sense, our findings suggest that not all minerals with theoretical vascular relevance show measurable independent associations with arterial stiffness progression in population-based longitudinal analyses.

From a dietary guidance perspective, the iron result should not be overinterpreted as supporting any change in clinical recommendations regarding iron intake in the general population. Current nutritional guidance continues to emphasize adequate intake according to age and sex, particularly because both deficiency and excess may have adverse health consequences. Our data simply suggest that the relationship between iron intake and vascular aging deserves further investigation in studies incorporating biomarkers of iron metabolism and a more detailed characterization of dietary iron sources.

### 4.4. Lack of Associations for Other Nutrients

An important overall result of the study is that most macronutrients and minerals were not independently associated with arterial stiffness after full adjustment. This pattern suggests that some associations observed in minimally adjusted models may be explained by confounding from body mass index, blood pressure, metabolic profile, or lifestyle behaviors. It also supports the idea that arterial stiffness is the result of a complex interaction of dietary and non-dietary exposures rather than the isolated effect of most single nutrients.

To contextualize our findings within standard dietary recommendations, it is important to consider the baseline intake levels of our cohort. The median fiber intake (25 g/day; IQR 19–30) was at the lower limit of current EFSA recommendations [62], which suggests at least 25–30 g/day, indicating that even modest increases toward recommended levels may provide vascular benefits. In contrast, median iron intake (16 mg/day; IQR 13–19) exceeded standard EFSA requirements (8 mg/day) [62], which may partly contextualize the observed association with arterial stiffness progression. Finally, the adverse vascular associations observed for alcohol are consistent with current public health recommendations emphasizing that no level of alcohol consumption can be considered completely safe for cardiovascular health.

At a broader level, these findings reinforce the notion that dietary effects on vascular aging may be cumulative, context-dependent, and more accurately represented by overall dietary patterns than by isolated nutrients. Nutrients do not operate in isolation in real-world diets, and their observed associations may depend on food sources, dietary combinations, and long-term behavioral patterns. In this context, the fact that only fiber, alcohol, carbohydrate intake, and iron retained associations after comprehensive adjustment suggests that these components may either exert more specific vascular effects or serve as indicators of broader dietary exposures relevant to vascular aging.

### 4.5. Clinical Implications

From a clinical and public health perspective, translating these findings into practical dietary modifications is particularly relevant. As observed in our study, a modest increase of 10 g/day in dietary fiber intake—equivalent to adding two servings of fruit or a portion of legumes to the daily diet—was associated with a measurable attenuation in arterial stiffness progression. Conversely, an increase of 10 g/day in alcohol consumption, approximately equivalent to one standard drink, was associated with a worsening of arterial stiffness over follow-up. Similarly, relatively small increases in dietary iron intake, within ranges achievable in habitual diets, were associated with adverse changes in arterial stiffness.

Furthermore, it is crucial to acknowledge that while the associations between fiber, iron, alcohol, and arterial stiffness progression reached statistical significance, the overall magnitude of these individual effects is relatively small. Consequently, their isolated clinical relevance may be limited. These specific nutritional components should not be viewed as standalone therapeutic targets, but rather as modest contributing factors that must be integrated into a broader, healthy global dietary pattern to achieve meaningful cardiovascular protection.

These findings are consistent with current recommendations emphasizing overall dietary quality rather than isolated nutrient modification. The inverse association observed for dietary fiber supports guidance promoting fruit, vegetables, legumes, and whole grains as part of cardiovascular prevention, whereas the positive association observed for alcohol aligns with current caution regarding habitual alcohol intake in vascular health. By contrast, the findings related to iron intake should be interpreted cautiously and should not be translated into specific dietary restriction recommendations, although they highlight the need for further research on the role of iron metabolism in vascular aging.

These results may be particularly relevant in primary care settings, where prevention strategies are often targeted at individuals without established cardiovascular disease but who may already exhibit early vascular alterations. In this context, arterial stiffness may serve as an intermediate phenotype reflecting the cumulative effect of unfavorable exposures before the onset of overt cardiovascular events.

### 4.6. Strengths and Limitations

This study has several strengths, including its longitudinal design, the population-based recruitment strategy, the simultaneous assessment of central and peripheral arterial stiffness using cfPWV and CAVI, and the use of progressively adjusted multivariable models [14,15,16,17]. These features provide a more comprehensive evaluation of vascular aging and strengthen the relevance of the findings in adults without previous cardiovascular disease.

Several limitations should also be considered. First, because this was an observational study, causal inferences cannot be established and residual confounding cannot be completely excluded. Second, dietary intake was assessed using a 3-day food record collected only at baseline. Although this method was based on a validated tool and was designed to include two weekdays and one weekend day, it remains subject to self-reporting bias and may not fully capture usual long-term dietary habits. In addition, because dietary intake was not reassessed at follow-up, we were unable to account for possible changes in eating behavior over the 5-year period. Third, the nutritional database used in the EVIDENT application did not allow detailed differentiation of several relevant nutrient subtypes and food-related characteristics [44]. Therefore, we could not distinguish simple from complex carbohydrates, soluble from insoluble fiber, heme from non-heme iron, or *n*-3 from *n*-6 polyunsaturated fatty acids. Similarly, the characterization of alcohol intake was limited, as we could not fully capture specific consumption patterns or reliably differentiate among all types of alcoholic beverages (e.g., fermented versus distilled beverages). Furthermore, our analytical approach focused strictly on isolated macronutrients and minerals. Consequently, a notable limitation is our inability to identify the specific whole foods or broad dietary patterns (e.g., the specific food sources of fiber or iron) driving these associations, which would provide greater clinical translation. These constraints limit the mechanistic and translational interpretation of some of the observed associations. Fourth, the dietary assessment tool was designed to record foods and beverages, but not to systematically capture the use of vitamin or mineral supplements. As a result, total intake of some nutrients, particularly minerals such as iron, may have been underestimated, and we could not differentiate between nutrients derived from habitual food sources and those obtained through high-dose supplementation. Moreover, we did not have biochemical markers of nutrient status, iron stores, or antioxidant balance, which would have allowed a more precise interpretation of the biological significance of the dietary findings. Fifth, although our analytical strategy aimed to estimate the independent association of nutrient intake with arterial stiffness progression by adjusting for major cardiovascular risk factors, we cannot rule out a degree of overadjustment. Some covariates included in the fully adjusted models, such as blood pressure, adiposity, or lipid-related variables, may lie partly on the causal pathway between diet and vascular damage and could therefore act as mediators. Consequently, the magnitude of the observed associations may have been conservative. Sixth, as in any observational study, the possibility of residual confounding cannot be entirely excluded. Despite comprehensive adjustment for known cardiovascular risk factors, unmeasured or imprecisely measured variables—such as lifetime physical activity patterns, sleep quality, psychological stress, or detailed socioeconomic status—could partially influence the observed associations. Consequently, these findings should be interpreted with caution. Finally, because multiple dietary nutrients were evaluated without formal correction for multiple comparisons, we cannot entirely rule out that some observed associations might be due to chance (Type I error). Therefore, these specific findings should be interpreted with caution and considered hypothesis-generating for future targeted trials.

## 5. Conclusions

In this population-based cohort of adults without previous cardiovascular disease, higher dietary fiber intake was associated with a lower progression of central arterial stiffness assessed by cfPWV, whereas alcohol consumption and iron intake were associated with greater CAVI progression. Carbohydrate intake showed a very small positive association with this index. Overall, these associations were modest in magnitude, and most nutrients were not independently related to arterial stiffness after full adjustment.

Taken together, these findings suggest that the relationship between diet and arterial stiffness is complex, selective, and likely multifactorial. The observed associations should be interpreted cautiously, as they do not imply causality, but rather suggest potential pathways linking specific dietary components with vascular aging. Further longitudinal studies and interventional research are warranted to confirm these findings and to better understand the underlying biological mechanisms.

## Figures and Tables

**Table 1:** Characteristics of the study population overall and by sex.

Variable	Total (*n* = 466)	Men (*n* = 226)	Women (*n* = 240)	*p*-Value
Age (years)	55.96 ± 14.15	55.94 ± 14.19	55.98 ± 14.13	0.973
Alcohol (g/week)	0.00 (0.00–65.00)	30.00 (0.00–105.00)	0.00 (0.00–20.00)	<0.001
No alcohol consumption (n, %)	236 (50.60)	87 (38.50)	149 (62.10)	<0.001
Mediterranean diet score	7.17 ± 2.07	6.73 ± 1.98	7.58 ± 2.08	<0.001
Total physical activity (MET-min/week)	1537.50 (742.12–2772.00)	2106.00 (1386.00–4134.00)	1263.75 (693.00–2079.00)	<0.001
Sitting time (h/week)	42.14 ± 17.81	47.97 ± 16.54	36.66 ± 17.23	<0.001
Systolic BP (mmHg)	119.70 ± 17.76	125.73 ± 16.49	114.03 ± 17.06	<0.001
Diastolic BP (mmHg)	75.64 ± 10.00	77.68 ± 9.16	73.73 ± 10.39	<0.001
Pulse pressure (mmHg)	44.06 ± 12.32	48.05 ± 12.03	40.30 ± 11.40	<0.001
Heart rate (bpm)	68.72 ± 9.52	67.65 ± 9.99	69.73 ± 8.96	0.018
Total cholesterol (mg/dL)	194.97 ± 32.84	192.61 ± 33.09	197.20 ± 32.52	0.132
LDL cholesterol (mg/dL)	115.58 ± 29.51	117.60 ± 30.57	113.68 ± 28.42	0.153
Triglycerides (mg/dL)	89.00 (67.00–89.00)	100.50 (73.25–134.00)	80.00 (62.75–107.00)	<0.001
Glucose (mg/dL)	85.00 (79.00–93.00)	87.00 (80.00–95.00)	84.00 (78.00–90.00)	0.001
Weight (kg)	72.48 ± 13.85	79.73 ± 11.85	65.66 ± 12.02	<0.001
Height (cm)	165.06 ± 9.69	171.81 ± 7.26	158.70 ± 7.03	<0.001
Body mass index (kg/m^2^)	26.55 ± 4.22	26.99 ± 3.41	26.13 ± 4.82	0.027
Pulse wave velocity (m/s)	7.60 (6.50–9.10)	7.90 (6.60–10.10)	7.30 (6.40–8.70)	<0.001
ΔcfPWV (m/s)	1.14 ± 1.76	1.33 ± 1.75	0.96 ± 1.75	0.023
Mean CAVI	8.01 ± 1.46	8.16 ± 1.50	7.87 ± 1.40	0.030
ΔCAVI	0.18 ± 0.89	0.24 ± 0.86	0.12 ± 0.92	0.129

Values are presented as mean ± standard deviation (SD) for approximately normally distributed variables, and as median (interquartile range, IQR) for non-normally distributed variables. The median alcohol intake for the total population was 0.00 g/week due to the high proportion of non-drinkers (>50%; 63% of women and 38.5% of men) in the overall cohort. Normality was assessed using the Shapiro–Wilk test and distribution shape (skewness/kurtosis). Between-group comparisons were performed using Student’s *t*-test for independent samples (Welch’s correction when variances were unequal based on Levene’s test) or the Mann–Whitney U test, as appropriate. A *p*-value < 0.05 was considered statistically significant. Abbreviations: BP, blood pressure; CAVI, cardio-ankle vascular index; cfPWV, carotid–femoral pulse wave velocity; MET, metabolic equivalent.

**Table 2:** Macronutrient intake overall and by sex.

Variable	Total (*n* = 466)	Men (*n* = 226)	Women (*n* = 240)	*p*-Value
Energy intake (kcal/day)	2061 (1770–24,766)	2177 (1812–2557)	1958 (1714–2324)	<0.001
Protein (g/day)	96 (80–111)	99 (85–115)	91 (76–108)	<0.001
Proteins (% of total energy)	18.44 ± 2.77	18.56 ± 3.00	18.33 ± 2.53	0.094
Carbohydrates (g/day)	193 (159–236)	203 (162–242)	188 (156–233)	0.059
Carbohydrates (% of total energy)	37.74 ± 6.73	37.31 ± 6.87	38.13 ± 6.59	0.293
Dietary fiber (g/day)	25 (19–30)	25 (19–30)	25 (19–31)	0.970
Total fat (g/day)	92 (77–114)	98 (81–117)	88 (75–109)	<0.001
Total fats (% of total energy)	40.98 ± 5.83	41.13 ± 5.79	40.84 ± 5.87	0.185
Saturated fatty acids (g/day)	30 (24–37)	32 (26–38)	28 (22–35)	0.002
Monounsaturated fatty acids (g/day)	43 (35–54)	47 (38–56)	40 (34–51)	<0.001
Polyunsaturated fatty acids (g/day)	16 (9–15)	12 (9–15)	11 (9–14)	0.093
Dietary cholesterol (mg/day)	358 (287–439)	394 (307–470)	328 (268–402)	<0.001
Alcohol (g/day)	0.00 (0.00–4.62)	0.37 (0.00–7.55)	0.00 (0.00–2.31)	<0.001
Water (mL/day)	1358 (1150–1606)	1357 (1148–1666)	1358 (1150–1562)	0.217

Values are presented as mean ± standard deviation (SD) for normally distributed variables and as median (interquartile range, IQR) for non-normally distributed variables. Between-group comparisons were performed using Student’s *t*-test or the Mann–Whitney U test, as appropriate. Multiple comparisons were corrected using the Benjamini–Hochberg false discovery rate (FDR) method. Effect sizes are reported as Cohen’s d (parametric) or r (non-parametric).

**Table 3:** Mineral intake overall and by sex.

Variable	Total (*n* = 466)	Men (*n* = 226)	Women (*n* = 240)	*p*-Value
Iron (mg/day)	15.7 (12.8–18.8)	16.2 (13.4–19.7)	15.4 (12.2–18.3)	0.417
Iodine (µg/day)	103 (80–135)	109 (82–141)	100 (79–128)	0.024
Magnesium (mg/day)	319 (261–375)	321 (270–376)	317 (257–373)	0.422
Zinc (mg/day)	11 (9–13)	12 (9–14)	10 (9–12)	0.006
Selenium (µg/day)	118 (94–151)	122 (97–163)	116 (90–142)	0.008
Sodium (mg/day)	3384 (2509–4335)	3669 (2843–4673)	3137 (2355–4108)	<0.001
Potassium (mg/day)	3555 (2934–4261)	3607 (2979–4345)	3523 (2914–4170)	0.146

Values are presented as mean ± standard deviation (SD) for normally distributed variables and as median (interquartile range, IQR) for non-normally distributed variables. Between-group comparisons were performed using Student’s *t*-test or the Mann–Whitney U test, as appropriate. Multiple comparisons were corrected using the Benjamini–Hochberg false discovery rate (FDR) method. Effect sizes are reported as Cohen’s d (parametric) or r (non-parametric).

**Table 4:** Summary of significant independent associations between dietary intake and 5-year arterial stiffness progression (fully adjusted models).

Nutrient Intake	Arterial Stiffness Index	Unstandardized β (95% CI)	*p*-Value
Dietary Fiber (g/day)	cfPWV progression (m/s)	−0.025 (−0.046 to −0.005)	0.018
Iron (mg/day)	CAVI progression	0.022 (0.004 to 0.041)	0.018
Alcohol (g/day)	CAVI progression	0.020 (0.006 to 0.034)	0.005

Models were fully adjusted for age, sex, baseline arterial stiffness, total energy intake, Mediterranean diet adherence score, physical activity, smoking status, body mass index, pulse pressure, heart rate, LDL cholesterol, and fasting glucose.

## Data Availability

The variables that we use in the analyses carried out to obtain the results of this work are available upon reasonable request to the corresponding author.

## Supplementary Materials

The following supporting information can be downloaded at https://www.mdpi.com/article/10.3390/nu18091314/s1, Figure S1: Participant recruitment flowchart; Figure S2: Five-year follow-up and attrition flowchart; Table S1: STROBE checklist; Table S2. Normality assessment of continuous variables; Table S3. Effect size estimates for sex differences (with 95% confidence intervals); Table S4. Normality assessment of macronutrient variables; Table S5. Effect sizes for sex differences in macronutrient intake (95% CI); Table S6. normality assessment of mineral variables; Table S7. Effect sizes for sex differences in mineral intake (95% CI).

## Abbreviations

The following abbreviations are used in this manuscript:

APISAL	Primary Care Research Unit of Salamanca
AS	Arterial stiffness
BMI	Body mass index
BP	Blood pressure
CAVI	Cardio-ankle vascular index
cfPWV	Carotid–femoral pulse wave velocity
CI	Confidence interval
EFSA	European Food Safety Authority
EVA	Early vascular aging
FDR	False discovery rate
IBSAL	Institute of Biomedical Research of Salamanca
IPAQ-SF	International Physical Activity Questionnaire–Short Form
IQR	Interquartile range
LDL	Low-density lipoprotein
MEDAS	Mediterranean Diet Adherence Screener
MET	Metabolic equivalent of task
MUFA	Monounsaturated fatty acids
PWV	Pulse wave velocity
REDIAPP	Primary Care Research Group of Castilla y León
RICAPPS	Research Network on Chronicity, Primary Care and Health Promotion
SD	Standard deviation
STROBE	Strengthening the Reporting of Observational Studies in Epidemiology
VIF	Variance inflation factor
WHO	World Health Organization

## References

[1] Roth G.A., Mensah G.A., Johnson C.O., Addolorato G., Ammirati E., Baddour L.M., Barengo N.C., Beaton A.Z., Benjamin E.J., Benziger C.P. (2020). Global Burden of Cardiovascular Diseases and Risk Factors, 1990–2019: Update From the GBD 2019 Study. J. Am. Coll. Cardiol..

[2] Nilsson P.M. (2008). Early Vascular Aging (EVA): Consequences and Prevention. Vasc. Health Risk Manag..

[3] Zhong Q., Hu M.-J., Cui Y.-J., Liang L., Zhou M.-M., Yang Y.-W., Huang F. (2018). Carotid–Femoral Pulse Wave Velocity in the Prediction of Cardiovascular Events and Mortality: An Updated Systematic Review and Meta-Analysis. Angiology.

[4] Sequí-Domínguez I., Cavero-Redondo I., Álvarez-Bueno C., Pozuelo-Carrascosa D.P., Nuñez de Arenas-Arroyo S., Martínez-Vizcaíno V. (2020). Accuracy of Pulse Wave Velocity Predicting Cardiovascular and All-Cause Mortality. A Systematic Review and Meta-Analysis. J. Clin. Med..

[5] Segers P., Rietzschel E.R., Chirinos J.A. (2020). How to Measure Arterial Stiffness in Humans. Arterioscler. Thromb. Vasc. Biol..

[6] Wilkinson I.B., Mäki-Petäjä K.M., Mitchell G.F. (2020). Uses of Arterial Stiffness in Clinical Practice. Arterioscler. Thromb. Vasc. Biol..

[7] Townsend R.R., Wilkinson I.B., Schiffrin E.L., Avolio A.P., Chirinos J.A., Cockcroft J.R., Heffernan K.S., Lakatta E.G., McEniery C.M., Mitchell G.F. (2015). Recommendations for Improving and Standardizing Vascular Research on Arterial Stiffness. Hypertension.

[8] Sun C.-K. (2013). Cardio-Ankle Vascular Index (CAVI) as an Indicator of Arterial Stiffness. Integr. Blood Press. Control.

[9] Shirai K., Hiruta N., Song M., Kurosu T., Suzuki J., Tomaru T., Miyashita Y., Saiki A., Takahashi M., Suzuki K. (2011). Cardio-Ankle Vascular Index (CAVI) as a Novel Indicator of Arterial Stiffness: Theory, Evidence and Perspectives. J. Atheroscler. Thromb..

[10] Miyoshi T., Ito H., Shirai K., Horinaka S., Higaki J., Yamamura S., Saiki A., Takahashi M., Masaki M., Okura T. (2021). Predictive Value of the Cardio-Ankle Vascular Index for Cardiovascular Events in Patients at Cardiovascular Risk. J. Am. Heart Assoc..

[11] Pase M.P., Grima N.A., Sarris J. (2011). The Effects of Dietary and Nutrient Interventions on Arterial Stiffness: A Systematic Review. Am. J. Clin. Nutr..

[12] Ravera A., Carubelli V., Sciatti E., Bonadei I., Gorga E., Cani D., Vizzardi E., Metra M., Lombardi C. (2016). Nutrition and Cardiovascular Disease: Finding the Perfect Recipe for Cardiovascular Health. Nutrients.

[13] Stanek A., Grygiel-Górniak B., Brożyna-Tkaczyk K., Myśliński W., Cholewka A., Zolghadri S. (2023). The Influence of Dietary Interventions on Arterial Stiffness in Overweight and Obese Subjects. Nutrients.

[14] Saz-Lara A., Cavero-Redondo I., Pascual-Morena C., Martínez-García I., Rodríguez-Gutiérrez E., Lucerón-Lucas-Torres M., Bizzozero-Peroni B., Moreno-Herráiz N., Martínez-Rodrigo A. (2023). Early Vascular Aging as an Index of Cardiovascular Risk in Healthy Adults: Confirmatory Factor Analysis from the EVasCu Study. Cardiovasc. Diabetol..

[15] García-Ortiz L., Recio-Rodríguez J.I., Rodríguez-Sánchez E., Patino-Alonso M.C., Agudo-Conde C., Rodríguez-Martín C., Castaño-Sánchez C., Runkle I., Gómez-Marcos M.A. (2012). Sodium and Potassium Intake Present a J-Shaped Relationship with Arterial Stiffness and Carotid Intima-Media Thickness. Atherosclerosis.

[16] GómezSánchez M., Gómez Sánchez L., Patino-Alonso M.C., Alonso-Domínguez R., Sánchez-Aguadero N., Lugones-Sánchez C., Rodríguez Sánchez E., García Ortiz L., Gómez-Marcos M.A. (2020). Adherence to the Mediterranean Diet in Spanish Population and Its Relationship with Early Vascular Aging According to Sex and Age: EVA Study. Nutrients.

[17] Gomez-Sanchez M., Gomez-Sanchez L., Patino-Alonso M.C., Cunha P.G., Recio-Rodriguez J.I., Alonso-Dominguez R., Sanchez-Aguadero N., Rodriguez-Sanchez E., Maderuelo-Fernandez J.A., Garcia-Ortiz L. (2020). Vascular Aging and Its Relationship with Lifestyles and Other Risk Factors in the General Spanish Population: Early Vascular Ageing Study. J. Hypertens..

[18] Tisdel D.M., Gadberry J.J., Burke S.L., Carlini N.A., Fleenor B.S., Campbell M.S. (2021). Dietary Fat and Alcohol in the Prediction of Indices of Vascular Health among Young Adults. Nutrition.

[19] Chan H.-T., Chan Y.-H., Yiu K.H., Li S.-W., Tam S., Lau C.-P., Tse H.-F. (2014). Worsened Arterial Stiffness in High-Risk Cardiovascular Patients with High Habitual Carbohydrate Intake: A Cross-Sectional Vascular Function Study. BMC Cardiovasc. Disord..

[20] Arnberg K., Larnkjær L., Michaelsen K.F., Mølgaard C. (2012). Central Adiposity and Protein Intake Are Associated with Arterial Stiffness in Overweight Children. J. Nutr..

[21] van de Laar R.J., Stehouwer C.D.A., van Bussel B.C., te Velde S.J., Prins M.H., Twisk J.W., Ferreira I. (2012). Lower Lifetime Dietary Fiber Intake Is Associated with Carotid Artery Stiffness: The Amsterdam Growth and Health Longitudinal Study. Am. J. Clin. Nutr..

[22] Campbell M.S., Fleenor B.S. (2018). Whole Grain Consumption Is Negatively Correlated with Obesity-Associated Aortic Stiffness: A Hypothesis. Nutrition.

[23] Anderson J.W., Baird P., Davis R.H., Ferreri S., Knudtson M., Koraym A., Waters V., Williams C.L. (2009). Health Benefits of Dietary Fiber. Nutr. Rev..

[24] Makki K., Deehan E.C., Walter J., Bäckhed F. (2018). The Impact of Dietary Fiber on Gut Microbiota in Host Health and Disease. Cell Host Microbe.

[25] Veronese N., Solmi M., Caruso M.G., Giannelli G., Osella A.R., Evangelou E., Maggi S., Fontana L., Stubbs B., Tzoulaki I. (2018). Dietary Fiber and Health Outcomes: An Umbrella Review of Systematic Reviews and Meta-Analyses. Am. J. Clin. Nutr..

[26] Ha J.Y., Kim M.K., Kang S., Nam J.S., Ahn C.W., Kim K.R., Park J.S. (2016). Serum Ferritin Levels Are Associated with Arterial Stiffness in Healthy Korean Adults. Vasc. Med..

[27] Kiechl S., Willeit J., Egger G., Poewe W., Oberhollenzer F. (1997). Body Iron Stores and the Risk of Carotid Atherosclerosis: Prospective Results from the Bruneck Study. Circulation.

[28] Yan F., Li K., Xing W., Dong M., Yi M., Zhang H. (2022). Role of Iron-Related Oxidative Stress and Mitochondrial Dysfunction in Cardiovascular Diseases. Oxid. Med. Cell. Longev..

[29] Sawicki K.T., De Jesus A., Ardehali H. (2023). Iron Metabolism in Cardiovascular Disease: Physiology, Mechanisms, and Therapeutic Targets. Circ. Res..

[30] Salonen J.T., Nyyssönen K., Korpela H., Tuomilehto J., Seppänen R., Salonen R. (1992). High Stored Iron Levels Are Associated with Excess Risk of Myocardial Infarction in Eastern Finnish Men. Circulation.

[31] Matek Sarić M., Sorić T., Juko Kasap Ž., Lisica Šikić N., Mavar M., Andruškienė J., Sarić A. (2025). Magnesium: Health Effects, Deficiency Burden, and Future Public Health Directions. Nutrients.

[32] Nielsen F.H. (2024). The Role of Dietary Magnesium in Cardiovascular Disease. Nutrients.

[33] Ronksley P.E., Brien S.E., Turner B.J., Mukamal K.J., Ghali W.A. (2011). Association of Alcohol Consumption with Selected Cardiovascular Disease Outcomes: A Systematic Review and Meta-Analysis. BMJ.

[34] Roerecke M., Tobe S.W., Kaczorowski J., Bacon S.L., Vafaei A., Hasan O.S.M., Krishnan R.J., Raifu A.O., Rehm J. (2018). Sex-Specific Associations Between Alcohol Consumption and Incidence of Hypertension: A Systematic Review and Meta-Analysis of Cohort Studies. J. Am. Heart Assoc..

[35] Piano M.R. (2017). Alcohol’s Effects on the Cardiovascular System. Alcohol Res..

[36] Del Giorno R., Maddalena A., Bassetti S., Gabutti L. (2022). Association between Alcohol Intake and Arterial Stiffness in Healthy Adults: A Systematic Review. Nutrients.

[37] Wood A.M., Kaptoge S., Butterworth A.S., Willeit P., Warnakula S., Bolton T., Paige E., Paul D.S., Sweeting M., Burgess S. (2018). Risk Thresholds for Alcohol Consumption: Combined Analysis of Individual-Participant Data for 599 912 Current Drinkers in 83 Prospective Studies. Lancet.

[38] Alonso-Diaz J., Gómez-Sánchez M., Sánchez-Moreno A., Lugones-Sánchez C., Rodriguez-Sanchez E., Garcia-Ortiz L., Gómez-Sánchez L., Gómez-Marcos M.A., EVA Investigators Group (2026). Association Between Increased Central and Peripheral Arterial 2 Stiffness and Vitamin Intake in Healthy Adults: EVA Follow-Up 3 Study. Nutrients.

[39] Gomez-Marcos M.A., Martinez-Salgado C., Gonzalez-Sarmiento R., Hernandez-Rivas J.M., Sanchez-Fernandez P.L., Recio-Rodriguez J.I., Rodriguez-Sanchez E., García-Ortiz L. (2016). Association between Different Risk Factors and Vascular Accelerated Ageing (EVA Study): Study Protocol for a Cross-Sectional, Descriptive Observational Study. BMJ Open.

[40] Vandenbroucke J.P., von Elm E., Altman D.G., Gøtzsche P.C., Mulrow C.D., Pocock S.J., Poole C., Schlesselman J.J., Egger M. (2014). Strengthening the Reporting of Observational Studies in Epidemiology (STROBE): Explanation and Elaboration. Int. J. Surg..

[41] World Medical Association (2025). World Medical Association Declaration of Helsinki. JAMA.

[42] (2010). The Reference Values for Arterial Stiffness’ Collaboration. Determinants of Pulse Wave Velocity in Healthy People and in the Presence of Cardiovascular Risk Factors: ‘Establishing Normal and Reference Values’. Eur. Heart J..

[43] Shirai K., Utino J., Otsuka K., Takata M. (2006). A Novel Blood Pressure-Independent Arterial Wall Stiffness Parameter; Cardio-Ankle Vascular Index (CAVI). J. Atheroscler. Thromb..

[44] Recio-Rodriguez J.I., Rodriguez-Martin C., Gonzalez-Sanchez J., Rodriguez-Sanchez E., Martin-Borras C., Martínez-Vizcaino V., Arietaleanizbeaskoa M.S., Magdalena-Gonzalez O., Fernandez-Alonso C., Maderuelo-Fernandez J.A. (2019). EVIDENT Smartphone App, a New Method for the Dietary Record: Comparison With a Food Frequency Questionnaire. JMIR Mhealth Uhealth.

[45] Schröder H., Fitó M., Estruch R., Martínez-González M.A., Corella D., Salas-Salvadó J., Lamuela-Raventós R., Ros E., Salaverría I., Fiol M. (2011). A Short Screener Is Valid for Assessing Mediterranean Diet Adherence among Older Spanish Men and Women. J. Nutr..

[46] Marshall A.L., Miller Y.D., Burton N.W., Brown W.J. (2010). Measuring Total and Domain-Specific Sitting. Med. Sci. Sports Exerc..

[47] Chu A., Ng S., Koh D., Müller-Riemenschneider F. (2018). Domain-Specific Adult Sedentary Behaviour Questionnaire (ASBQ) and the GPAQ Single-Item Question: A Reliability and Validity Study in an Asian Population. Int. J. Environ. Res. Public Health.

[48] Lee P.H., Macfarlane D.J., Lam T., Stewart S.M. (2011). Validity of the International Physical Activity Questionnaire Short Form (IPAQ-SF): A Systematic Review. Int. J. Behav. Nutr. Phys. Act..

[49] Meh K., Jurak G., Sorić M., Rocha P., Sember V. (2021). Validity and Reliability of IPAQ-SF and GPAQ for Assessing Sedentary Behaviour in Adults in the European Union: A Systematic Review and Meta-Analysis. Int. J. Environ. Res. Public Health.

[50] Williams B., Mancia G., Spiering W., Agabiti Rosei E., Azizi M., Burnier M., Clement D.L., Coca A., de Simone G., Dominiczak A. (2018). 2018 ESC/ESH Guidelines for the Management of Arterial Hypertension. Eur. Heart J..

[51] Threapleton D.E., Greenwood D.C., Evans C.E.L., Cleghorn C.L., Nykjaer C., Woodhead C., Cade J.E., Gale C.P., Burley V.J. (2013). Dietary Fibre Intake and Risk of Cardiovascular Disease: Systematic Review and Meta-Analysis. BMJ.

[52] Kim Y., Je Y. (2016). Dietary Fibre Intake and Mortality from Cardiovascular Disease and All Cancers: A Meta-Analysis of Prospective Cohort Studies. Arch. Cardiovasc. Dis..

[53] Demirci B.G., Tutal E., Eminsoy I.O., Kulah E., Sezer S. (2019). Dietary Fiber Intake: Its Relation With Glycation End Products and Arterial Stiffness in End-Stage Renal Disease Patients. J. Ren. Nutr..

[54] Reynolds A., Mann J., Cummings J., Winter N., Mete E., Te Morenga L. (2019). Carbohydrate Quality and Human Health: A Series of Systematic Reviews and Meta-Analyses. Lancet.

[55] Bhupathiraju S.N., Tobias D.K., Malik V.S., Pan A., Hruby A., Manson J.E., Willett W.C., Hu F.B. (2014). Glycemic Index, Glycemic Load, and Risk of Type 2 Diabetes: Results from 3 Large US Cohorts and an Updated Meta-Analysis. Am. J. Clin. Nutr..

[56] Seidelmann S.B., Claggett B., Cheng S., Henglin M., Shah A., Steffen L.M., Folsom A.R., Rimm E.B., Willett W.C., Solomon S.D. (2018). Dietary Carbohydrate Intake and Mortality: A Prospective Cohort Study and Meta-Analysis. Lancet Public Health.

[57] Briasoulis A., Agarwal V., Messerli F.H. (2012). Alcohol Consumption and the Risk of Hypertension in Men and Women: A Systematic Review and Meta-Analysis. J. Clin. Hypertens..

[58] O’Neill D., Britton A., Brunner E.J., Bell S. (2017). Twenty-Five-Year Alcohol Consumption Trajectories and Their Association With Arterial Aging: A Prospective Cohort Study. J. Am. Heart Assoc..

[59] Vinchi F., Muckenthaler M.U., Da Silva M.C., Balla G., Balla J., Jeney V. (2014). Atherogenesis and Iron: From Epidemiology to Cellular Level. Front. Pharmacol..

[60] D’Elia L., Galletti F., La Fata E., Sabino P., Strazzullo P. (2018). Effect of Dietary Sodium Restriction on Arterial Stiffness. J. Hypertens..

[61] Ishida A., Isotani A., Fujisawa M., del Saz E.G., Okumiya K., Kimura Y., Manuaba I.I.B., Rantetampang A.L., Ohya Y., Matsubayashi K. (2021). Effects of a Low-Salt and High-Potassium Diet on Arterial Stiffness and Left Ventricular Function in Indigenous Papuans. J. Am. Heart Assoc..

[62] EFSA (European Food Safety Authority) Dietary Reference Values for the EU Finder. https://www.efsa.europa.eu/en/topics/topic/dietary-reference-values.

