# Supplementary material for: Dietary Fiber Is Inversely Associated with Central Arterial Stiffness Progression, While Alcohol and Iron Intake Are Positively Associated with CAVI: A 5-Year Longitudinal Study
Source: Nutrients. 2026 Apr 22;18(9):1314. doi: 10.3390/nu18091314 (PMC13164730; doi:10.3390/nu18091314)
Supplement: Supplementary file 1 [file nutrients-18-01314-s001.zip › nutrients-4236666-supplementary.pdf]

Supplementary Materials:

The following supporting information consists on: Figure S1: Participant recruitment flowchart; Figure S2: Five-year follow-up and attrition flowchart; Table S1: STROBE checklist; Table S2. Normality Assessment of Continuous Variables; Table S3. Effect Size Estimates for Sex Differences (with 95% Confidence Intervals); Table S4. Normality Assessment of Macro-nutrient Variables; Table S5. Effect Sizes for Sex Differences in Macronutrient Intake (95% CI); Table S6. Normality Assessment of Minerals Variables; Table S7. Effect Sizes for Sex Differences in Minerals Intake (95% CI).

Appendix B. Supplementary material

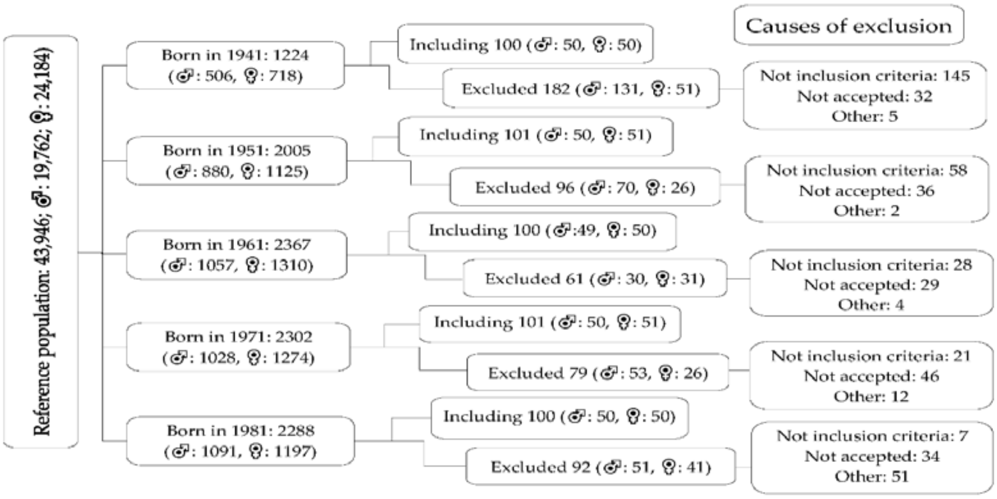

Figure S1. EVA study flowchart. Diagram showing the reference population by age group and sex, the number of subjects screened, included, and excluded, and the main reasons for exclusion. In total, 259 individuals did not meet the inclusion criteria, 177 declined participation, and 74 could not be contacted (change of address or telephone number). The replenishment rate was 35.4% and the response rate was 64.6%. Symbols: ♂, males; ♀, females.

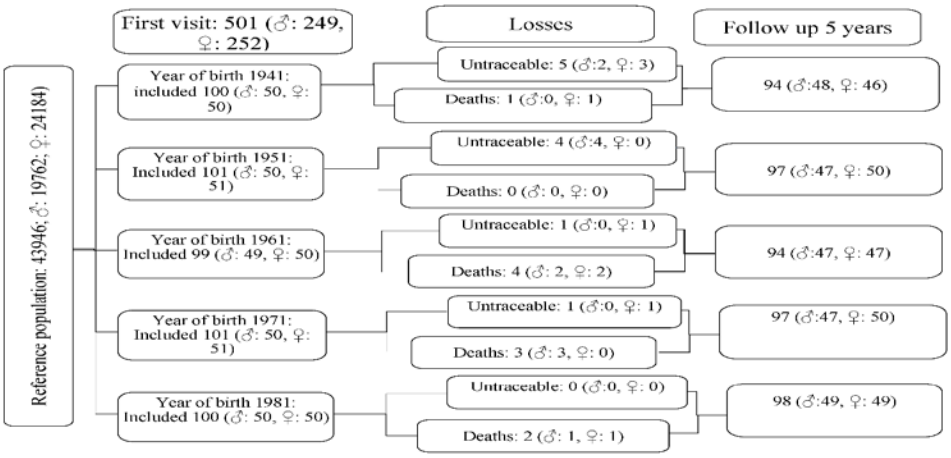

Figure S2. Flowchart of the EVA study follow-up phase. Diagram summarizing participant status over five years. During follow-up, 10 participants died (6 ♂; 4 ♀) and 11 could not be contacted (6 ♂; 5 ♀).

Table S1. STROBE Statement—checklist of items that should be included in reports of observational studies

	Item No	Recommendation	Page No
**Title and abstract**	1	(*a*) Indicate the study's design with a commonly used term in the title or the abstract	Title (page 1)
		(*b*) Provide in the abstract an informative and balanced summary of what was done and what was found	Abstract (pages 1–2)
**Introduction**			
Background/rationale	2	Explain the scientific background and rationale for the investigation being reported	Introduction (pages 3–4)
Objectives	3	State specific objectives, including any prespecified hypotheses	Introduction (last paragraph, pages 3–4)
**Methods**			
Study design	4	Present key elements of study design early in the paper	Methods section 2.1 (page 4)
Setting	5	Describe the setting, locations, and relevant dates, including periods of recruitment, exposure, follow-up, and data collection	Methods section 2.1 (page 4)
Participants	6	(*a*) *Cohort study*—Give the eligibility criteria, and the sources and methods of selection of participants. Describe methods of follow-up	Methods section 2.1 (page 4); Figures S1–S2

		Case-control study—Give the eligibility criteria, and the sources and methods of case ascertainment and control selection. Give the rationale for the choice of cases and controls<br>*Cross-sectional study*—Give the eligibility criteria, and the sources and methods of selection of participants	
		(*b*) *Cohort study*—For matched studies, give matching criteria and number of exposed and unexposed<br>*Case-control study*—For matched studies, give matching criteria and the number of controls per case	Not applicable
Variables	7	Clearly define all outcomes, exposures, predictors, potential confounders, and effect modifiers. Give diagnostic criteria, if applicable	Methods sections 2.2–2.4 (pages 5–6)
Data sources/ measurement	8*	For each variable of interest, give sources of data and details of methods of assessment (measurement). Describe comparability of assessment methods if there is more than one group	Methods sections 2.2–2.3 (pages 5–6)
Bias	9	Describe any efforts to address potential sources of bias	Statistical analysis (pages 6–7); Discussion section 4.6 (page 18)
Study size	10	Explain how the study size was arrived at	Methods section 2.1 (page 4)
Quantitative variables	11	Explain how quantitative variables were handled in the analyses. If applicable, describe which groupings were chosen and why	Statistical analysis (page 6)
Statistical methods	12	(*a*) Describe all statistical methods, including those used to control for confounding	Statistical analysis (pages 6–7)
		(*b*) Describe any methods used to examine subgroups and interactions	Not applicable
		(*c*) Explain how missing data were addressed	Methods section 2.1 (complete-case analysis); Discussion section 4.6 (page 18)
		(*d*) *Cohort study*—If applicable, explain how loss to follow-up was addressed<br>*Case-control study*—If applicable, explain how matching of cases and controls was addressed	Methods section 2.1 (page 4); Figures S1–S2

	Cross-sectional study—If applicable, describe analytical methods taking account of sampling strategy	
	(*e*) Describe any sensitivity analyses	Results section 3.5 (page 11)

Continued on next page

Results

Participants	13*	(a) Report numbers of individuals at each stage of study—eg numbers potentially eligible, examined for eligibility, confirmed eligible, included in the study, completing follow-up, and analysed	Methods section 2.1 (page 4); Figures S1–S2 (Supplementary Materials)
		(b) Give reasons for non-participation at each stage	Figures S1–S2 (Supplementary Materials)
		(c) Consider use of a flow diagram	Figures S1–S2 (Supplementary Materials)
Descriptive data	14*	(a) Give characteristics of study participants (eg demographic, clinical, social) and information on exposures and potential confounders	Results, Table 1 (page 7)
		(b) Indicate number of participants with missing data for each variable of interest	Methods section 2.1 (page 4)
		(c) *Cohort study*—Summarise follow-up time (eg, average and total amount)	Methods section 2.1 (page 4)
Outcome data	15*	*Cohort study*—Report numbers of outcome events or summary measures over time	Results sections 3.4–3.5 (pages 9–12)
		Case-control study—Report numbers in each exposure category, or summary measures of exposure	Not applicable
		Cross-sectional study—Report numbers of outcome events or summary measures	Not applicable
Main results	16	(*a*) Give unadjusted estimates and, if applicable, confounder-adjusted estimates and their precision (eg, 95% confidence interval). Make clear which confounders were adjusted for and why they were included	Results section 3.4–3.5 (pages 10–11)
		(*b*) Report category boundaries when continuous variables were categorized	Not applicable
		(*c*) If relevant, consider translating estimates of relative risk into absolute risk for a meaningful time period	Results section 3.4–3.5 (pages 10–11)
Other analyses	17	Report other analyses done—eg analyses of subgroups and interactions, and sensitivity analyses	Results section 3.5 (sensitivity analysis) (page 11)

Discussion

Key results	18	Summarise key results with reference to study objectives	Discussion section 4.1 (page 14)
Limitations	19	Discuss limitations of the study, taking into account sources of potential bias or imprecision. Discuss both direction and magnitude of any potential bias	Discussion section 4.6 (page 18)

Interpretation	20	Give a cautious overall interpretation of results considering objectives, limitations, multiplicity of analyses, results from similar studies, and other relevant evidence	Discussion (pages 14–17)
Generalisability	21	Discuss the generalisability (external validity) of the study results	Discussion (pages 16–17)
**Other information**			
Funding	22	Give the source of funding and the role of the funders for the present study and, if applicable, for the original study on which the present article is based	Funding section (page 19)

*Give information separately for cases and controls in case-control studies and, if applicable, for exposed and unexposed groups in cohort and cross-sectional studies.

Note: An Explanation and Elaboration article discusses each checklist item and gives methodological background and published examples of transparent reporting. The STROBE checklist is best used in conjunction with this article (freely available on the Web sites of PLoS Medicine at http://www.plosmedicine.org/ Annals of Internal Medicine at http://www.annals.org/, and Epidemiology at http://www.epidem.com/). Information on the STROBE Initiative is available at www.strobe-statement.org.

Table S2. Normality Assessment of Continuous Variables.

**Variable**	**Shapiro–Wilk (Total)**	**p**	**Shapiro–Wilk (Men)**	**p**	**Shapiro–Wilk (Women)**	**p**	**Skewness (Total)**	**Kurtosis (Total)**
Age (years)	<0.001		<0.001		<0.001		-0.0057	-1.2663
Alcohol (g/week)	<0.001		<0.001		<0.001		2.4576	7.4007
Mediterranean diet score	<0.001		<0.001		<0.001		0.0937	-0.278
Total physical activity (MET-min/week)	<0.001		<0.001		<0.001		3.9024	19.6888
Sitting time (h/week)	<0.001		0.0728		<0.001		0.3252	-0.1918
Systolic BP (mmHg)	<0.001		<0.001		<0.001		0.5941	0.6537
Diastolic BP (mmHg)	0.1410		0.0339		0.0568		0.2059	0.1333
Pulse pressure (mmHg)	<0.001		<0.001		<0.001		0.7805	0.5088
Heart rate (bpm)	0.0020		0.0045		0.1167		0.3692	0.6871
Total cholesterol (mg/dL)	0.8738		0.9978		0.1082		0.0904	-0.0314
LDL cholesterol (mg/dL)	0.6717		0.9904		0.1632		0.1461	0.1355
Glucose (mg/dL)	<0.001		<0.001		<0.001		3.7205	27.3711
Weight (kg)	<0.001		<0.001		<0.001		0.5226	0.6028

Height (cm)	0.0130	0.3617	0.1916	0.0235	-0.5631
Body mass index (kg/m²)	<0.001	<0.001	<0.001	0.5662	0.8602
Pulse wave velocity (m/s)	<0.001	<0.001	<0.001	1.4193	2.2292
ΔcfPWV (m/s)	<0.001	<0.001	<0.001	-0.3258	2.7153
Mean CAVI	<0.001	0.1291	<0.001	0.3827	-0.0087
ΔCAVI	<0.001	0.0176	<0.001	-0.1391	1.8532

Normality was assessed using the Shapiro–Wilk test. Skewness and kurtosis values correspond to the overall sample. A p-value < 0.05 indicates deviation from normal distribution.

Table S3. Effect Size Estimates for Sex Differences (with 95% Confidence Intervals).

Variable	Statistical Test	Effect Size (95% CI)
Age (years)	Mann–Whitney U	r = 0.007 [-0.084, 0.097]
Alcohol (g/week)	Mann–Whitney U	r = 0.320 [0.236, 0.399]
Mediterranean diet score	Mann–Whitney U	r = 0.203 [0.114, 0.288]
Total physical activity (MET-min/week)	Mann–Whitney U	r = 0.323 [0.240, 0.402]
Sitting time (h/week)	Mann–Whitney U	r = 0.328 [0.244, 0.407]
Pulse pressure (mmHg)	Mann–Whitney U	r = 0.343 [0.260, 0.420]
Heart rate (bpm)	Mann–Whitney U	r = 0.121 [0.031, 0.210]
Total cholesterol (mg/dL)	Student's t-test	d = -0.140 [-0.322, 0.042]
LDL cholesterol (mg/dL)	Student's t-test	d = 0.133 [-0.049, 0.316]
Glucose (mg/dL)	Mann–Whitney U	r = 0.152 [0.062, 0.240]
Weight (kg)	Mann–Whitney U	r = 0.536 [0.468, 0.597]
Height (cm)	Student's t-test	d = 1.836 [1.619, 2.052]
Body mass index (kg/m²)	Mann–Whitney U	r = 0.137 [0.046, 0.225]
Pulse wave velocity (m/s)	Mann–Whitney U	r = 0.158 [0.068, 0.245]
ΔcfPWV (m/s)	Mann–Whitney U	r = 0.110 [0.019, 0.198]
Mean CAVI	Mann–Whitney U	r = 0.112 [0.022, 0.201]
ΔCAVI	Mann–Whitney U	r = 0.102 [0.011, 0.191]

Table S4. Normality Assessment of Macronutrient Variables.

Variable	Shapiro–Wilk p (Total)	Skewness	Kurtosis	Distribution
Energy intake (kcal/day)	<0.001	0.763	1.300	Non-normal
Protein (g/day)	<0.001	0.850	2.028	Non-normal
Carbohydrates (g/day)	<0.001	0.704	0.744	Non-normal
Dietary fiber (g/day)	<0.001	0.526	0.086	Non-normal
Total fat (g/day)	<0.001	0.926	1.510	Non-normal
Saturated fatty acids (g/day)	<0.001	0.891	1.303	Non-normal

Monounsaturated fatty acids (g/day)	<0.001	0.917	1.268	Non-normal
Polyunsaturated fatty acids (g/day)	<0.001	1.081	1.381	Non-normal
Dietary cholesterol (mg/day)	<0.001	0.840	0.826	Non-normal
Alcohol (g/day)	<0.001	3.197	12.613	Non-normal
Water (mL/day)	0.003	0.368	0.814	Non-normal

Normality was assessed using the Shapiro–Wilk test. Positive skewness indicates right-tailed distribution.

Table S5. Effect Sizes for Sex Differences in Macronutrient Intake (95% CI).

Variable	Statistical Test	Effect Size (95% CI)
Energy intake (kcal/day)	Mann–Whitney U	r = 0.171 [0.081, 0.258]
Protein (g/day)	Mann–Whitney U	r = 0.182 [0.093, 0.269]
Carbohydrates (g/day)	Mann–Whitney U	r = 0.087 [-0.004, 0.177]
Dietary fiber (g/day)	Mann–Whitney U	r = 0.002 [-0.089, 0.093]
Total fat (g/day)	Mann–Whitney U	r = 0.168 [0.079, 0.255]
Saturated fatty acids (g/day)	Mann–Whitney U	r = 0.146 [0.056, 0.234]
Monounsaturated fatty acids (g/day)	Mann–Whitney U	r = 0.191 [0.102, 0.277]
Polyunsaturated fatty acids (g/day)	Mann–Whitney U	r = 0.078 [-0.013, 0.167]
Dietary cholesterol (mg/day)	Mann–Whitney U	r = 0.218 [0.129, 0.303]
Alcohol (g/day)	Mann–Whitney U	r = 0.205 [0.116, 0.290]
Water (mL/day)	Mann–Whitney U	r = 0.057 [-0.034, 0.147]

Effect sizes are presented as Cohen’s d for parametric comparisons and r for non-parametric comparisons. Confidence intervals were calculated using standard formulas.

Table S6. Normality Assessment of Minerals Variables.

Variable	Shapiro–Wilk p (Total)	Skewness	Kurtosis	Distribution
Iron (mg/day)	<0.001	0.739	0.973	Non-normal
Iodine (µg/day)	<0.001	2.075	8.473	Non-normal
Magnesium (mg/day)	<0.001	0.547	0.534	Non-normal
Zinc (mg/day)	<0.001	0.831	1.440	Non-normal
Selenium (µg/day)	<0.001	0.781	1.025	Non-normal
Sodium (mg/day)	<0.001	1.158	2.230	Non-normal
Potassium (mg/day)	0.008	0.295	0.246	Non-normal

Normality was assessed using the Shapiro–Wilk test. Positive skewness indicates right-tailed distribution.

Table S7. Effect Sizes for Sex Differences in Minerals Intake (95% CI).

Variable	Statistical Test	Effect Size (95% CI)
Iron (mg/day)	Mann–Whitney U	r = 0.110 [0.020, 0.199]
Iodine (μg/day)	Mann–Whitney U	r = 0.104 [0.014, 0.193]
Magnesium (mg/day)	Mann–Whitney U	r = 0.037 [-0.054, 0.128]
Zinc (mg/day)	Mann–Whitney U	r = 0.128 [0.037, 0.216]
Selenium (μg/day)	Mann–Whitney U	r = 0.122 [0.032, 0.211]
Sodium (mg/day)	Mann–Whitney U	r = 0.174 [0.084, 0.260]
Potassium (mg/day)	Mann–Whitney U	r = 0.067 [-0.024, 0.157]

Effect sizes are presented as Cohen's d for parametric comparisons and r for non-parametric comparisons. Confidence intervals were calculated using standard formulas.
